# Conductive printable electrodes tuned by boron-doped nanodiamond foil additives for nitroexplosive detection

**DOI:** 10.1007/s00604-022-05371-w

**Published:** 2022-07-05

**Authors:** Anna Dettlaff, Michał Rycewicz, Mateusz Ficek, Aleksandra Wieloszyńska, Mateusz Szala, Jacek Ryl, Robert Bogdanowicz

**Affiliations:** 1 Faculty of Chemistry, Department of Energy Conversion and Storage, Gdańsk University of Technology, 11/12 Narutowicza St, 80-233 Gdańsk, Poland; 2 Faculty of Electronics, Telecommunications and Informatics, Department of Metrology and Optoelectronics, Gdańsk University of Technology, 11/12 Narutowicza St, 80-233 Gdańsk, Poland; 3 Military University of Technology, S. Kaliskiego 2, 00-908 Warsaw, Poland; 4 Institute of Nanotechnology and Materials Engineering and Advanced Materials Center, Gdańsk University of Technology, 11/12 Narutowicza St, 80-233 Gdańsk, Poland

**Keywords:** TNT, PLA, Graphene filament, Diamond, DPV

## Abstract

An efficient additive manufacturing-based composite material fabrication for electrochemical applications is reported. The composite is composed of commercially available graphene-doped polylactide acid (G-PLA) 3D printouts and surface-functionalized with nanocrystalline boron-doped diamond foil (NDF) additives. The NDFs were synthesized on a tantalum substrate and transferred to the 3D-printout surface at 200 °C. No other electrode activation treatment was necessary. Different configurations of low- and heavy-boron doping NDFs were evaluated. The electrode kinetics was analyzed using electrochemical procedures: cyclic voltammetry and electrochemical impedance spectroscopy. The quasi-reversible electrochemical process was reported in each studied case. The studies allowed confirmation of the CV peak-to-peak separation of 63 mV and remarkably high heterogeneous electron transfer rate constant reaching 6.1 × 10^−2^ cm s^−1^ for 10 k ppm [B]/[C] thin NDF fitted topside at the G-PLA electrode. Differential pulse voltammetry was used for effective 2,4,6-trinitrotoluene (TNT) detection at the studied electrodes with a 87 ppb limit of detection, and wide linearity range between peak current density and the analyte concentration (0.064 to 64 ppm of TNT). The reported electrode kinetic differences originate primarily from the boron-dopant concentration in the diamond and the various contents of the non-diamond carbon phase.

**Graphical abstract:**

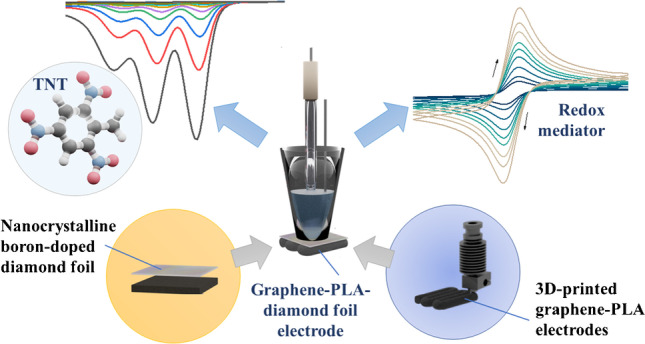

**Supplementary Information:**

The online version contains supplementary material available at 10.1007/s00604-022-05371-w.

## Introduction

The rapidly growing attention in the general culture around 3-dimensional (3D) printing technology over the past few years has mirrored its heightened attention of scientists. The 3D printing process provides the one-step formation of unique objects adapted to specific applications. Compare to conventional fabrication, 3D printing is a versatile, cost-effective technology that offers both fast prototyping and large-scale production.

One of the most popular 3D printing techniques, which is particularly noteworthy, is the Fused Filament Fabrication (FFF) process. FFF allows the use of a wide range of printing polymer materials such as poly(lactic acid) (PLA), thermoplastic elastomers (TPE), poly(ethylene terephthalate) (PET), acrylonitrile butadiene styrene (ABS), poly(vinyl alcohol) (PVA), poly(carbonate) (PC), and nylon [1]. Furthermore, the printing material can take the form of a wire, where the thermoplastic polymer plays role of a binder. Printing electrodes using carbon-based conductive filaments have been adopted in analytical chemistry and electrochemistry [2, 3]. In particular, commercially available carbon black/PLA [4–6], carbon black/ABS [7], and graphene/PLA [8–11] filaments have been used as a starting material for electrode applications in electrochemical sensors or biosensors. However, it should be emphasized that such electrodes suffer from the insulating nature of the PLA and ABS polymers [12]. Different methods have been used for the activation of printed electrodes. The surface polymer layer may be removed mechanically (polishing) [10], chemically (dissolution using solvents, saponification reaction [4, 13]), electrochemically [14], or by laser ablation [6]. Appropriately modified materials can find applications as temperature [15], strain [16, 17], or electrochemical sensors [18, 19]. Recently, Muñoz and Pumera realized a 3D-Printed Covid-19 immunosensor with electronic readout [20]. For this purpose, they anchored the COVID-19 protein on a 3D-printed graphene-based electrode surface. Moreover, Tang et al. [21] used a 3D printing method for cancer protein detection. Detection limits of 0.5 pg mL^−1^ were achieved. The all above systems provide fast results at low costs, which is a definite advantage.

In this work, we propose a simple method of printed graphene-based electrode electro-activation by modification using high-quality, free-standing boron-doped diamond foil for nitroexplosive detection. Nanocrystalline diamond foils (NDFs) are highly ordered diamond nanosheet arrays exhibiting excellent electrochemical properties [22]. Our prior preliminary work revealed an efficient method of fabrication of large area, thin NDFs revealing interesting electronic transfer properties along with efficient tunneling in the diamond-on-graphene junction configuration [23]. To the best of our knowledge, the literature does not report studies focused on the surface modification of printed graphene/poly(lactic acid) by nanodiamond foil.

The created electrode is very well suited as an electrode working in an electrochemical sensor used to detect nitroexplosive compounds in the aqueous environment. Highly energetic nitroaromatic-type materials are still widely used for military and mining purposes due to their low cost. Very large amounts of these compounds found their way into the environment during World War I and II. The best-known representative of this group of compounds is 2,4,6-trinitrotoluene (TNT), which also is classified by United States Environmental Protection Agency (EPA), e.g., as contaminants of emerging concern (CEC) due to its high toxicity and mutagenic effect on the environment and all life forms. To detect these compounds, expensive and time-consuming chromatographic analyses are most often used [24–26]. Hence, an inexpensive and fast way to carry out environmental monitoring to detect these compounds is essential.

## Material and methods

### Preparation of diamond foils

Nanodiamond foils (NDF) were synthesized using Microwave Plasma Enhanced Chemical Vapor Deposition (MPECVD). The details of the process can be found in our previous works [23, 27] and Supplemental Information. In this study, the boron doping level, expressed as the [B]/[C] ratio, was 500 ppm, and 10,000 ppm. The growth time did not exceed 300 min. The topography and structural composition of NDF are presented in [27].

### Preparation of the graphene-polymer electrodes

The graphene-PLA electrodes were designed in a CAD application and printed using a 3D printer (Ender 3 Pro, Creality). Printing parameters were optimized for the available printer. The nozzle diameter was 0.5 mm with temperature set at 220 °C, the bed temperature was 60 °C, and the print speed — 60 mm/s. The electrodes were printed horizontally with 100% infill density. We used a conductive graphene PLA filament from Black Magic 3D as the substrate for the NDF.

### Preparation of the composite graphene-polymer-nanodiamond foil electrodes

**Fig. 1:**
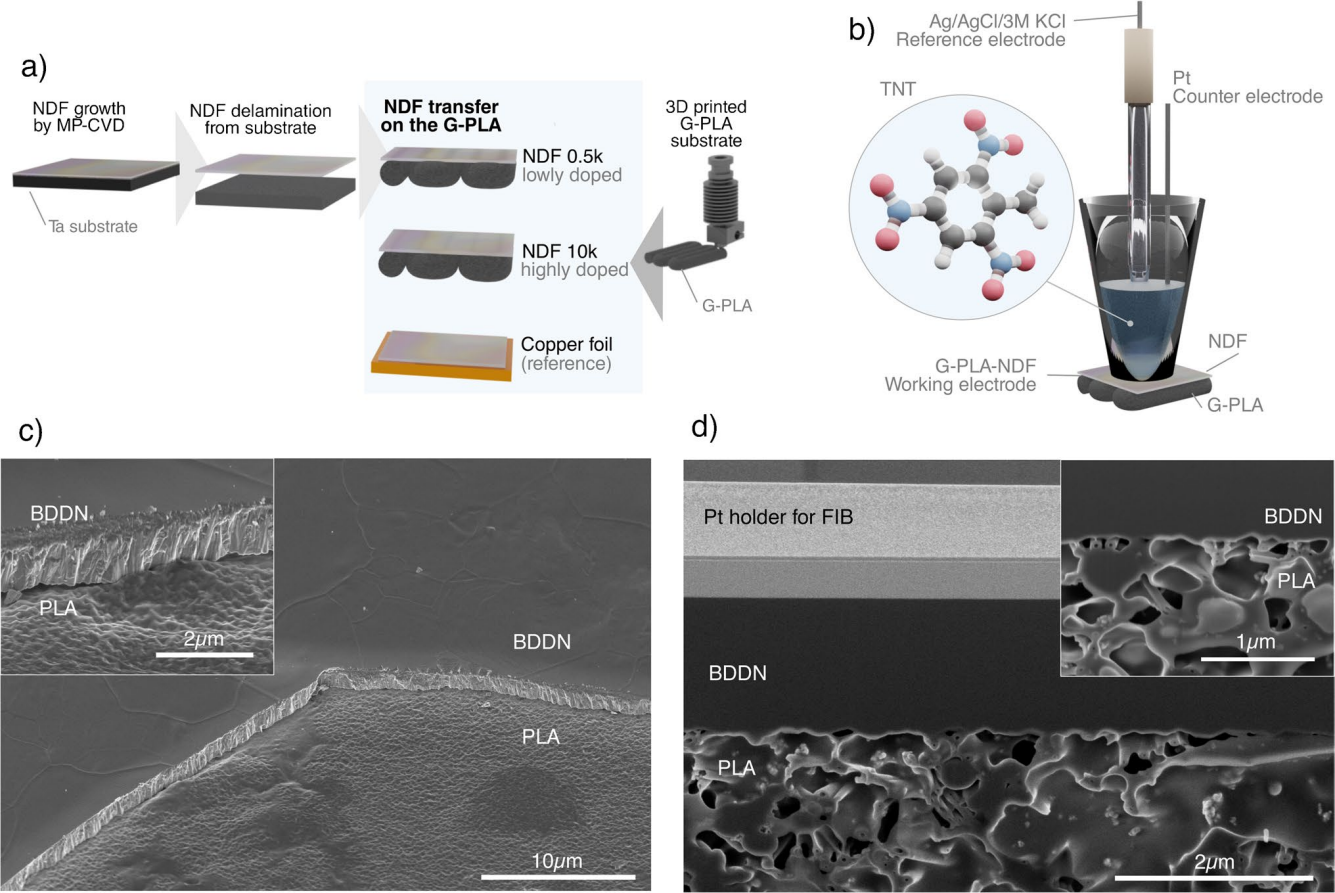
(**a**) Fabrication of G-PLA-NDF composite electrodes, (**b**) three-electrode electrochemical setup for 2,4,6-trinitrotoluene detection, (**c**) scanning electron microscopy image of the G-PLA-NDF-10 k-bottom electrode, and (**d**) cross section of the realized electrode (the nanodiamond foil is covering the graphene-polymer electrode)

As previously reported [23, 27], the nanodiamond foils showed low adhesion to the tantalum substrate, so they could be easily transferred to the printed graphene/poly(lactic acid) (G-PLA) electrode. The required temperature of the G-PLA electrode to transfer nanodiamond foil is 200 °C, which may vary depending on the printer filament. After reaching the required temperature for G-PLA, NDF was placed on the hot G-PLA surface utilizing tweezers and pressed by a tantalum substrate. It was then cooled down to room temperature. For our studies, we decided to use the top side (NDF-top), and the reverse side of nanodiamond foil (NDF-bottom) delaminated from the metal substrate with different levels of doping ([B]/[C] = 0.5 k ppm and 10 k ppm) on the G-PLA. As a result, we obtained four types of samples: G-PLA-NDF-10 k-top ([B]/[C] = 10,000 ppm, top side), G-PLA-NDF-10 k-bottom ([B]/[C] = 10,000 ppm, reverse side), G-PLA-NDF-0.5 k-top ([B]/[C] = 500 ppm, top side), and G-PLA-NDF-0.5 k-bottom ([B]/[C] = 500 ppm, reverse side). The procedure is presented in Fig. 1a. To compare the results, we also transferred nanodiamond foil to the commercially available copper tape with conductive acrylic adhesive (https://www.3m.com/3M/en_US/p/d/b00041302/) as a reference samples (Cu-NDF-10 k-bottom and Cu-NDF-10 k-top).

### Characterization techniques

The electrochemical measurements were done using a VMP-300 potentiostat–galvanostat (Bio-logic, France). The detection of 2,4,6-trinitrotoluene (TNT) was performed in a standard three-electrode electrochemical setup (Fig. 1b). We applied graphene-PLA-NDF composite as the working electrode and a Pt wire as the counter electrode. Ag|AgCl in 3 M KCl served as the reference electrode. Before the investigation, all solutions were deoxygenated with argon gas.

Cyclic voltammetry (CV) and electrochemical impedance spectroscopy (EIS) were used to investigate the kinetics of the electrodes. CV and EIS were conducted in 1 mM K_3_[Fe(CN)_6_]/1 mM K_4_[Fe(CN)_6_] redox couple containing 0.5 M Na_2_SO_4_. Cyclic voltammetry was conducted for chosen scan rates ν (5, 10, 25, 50, 75, 100, 150, 200, 300 mV s^−1^). EIS measurements were performed across a frequency range from 0.05 Hz to 100 kHz at a formal potential (held for 20 min) with six points per frequency decade and a perturbation amplitude equal to 10 mV.

Differential pulse voltammetry (DPV) was performed for TNT determination. The measurements were done in a 0.1 M phosphate buffer solution (PBS) of pH = 6.8 consisting of 0.064, 0.125, 0.25, 0.5, 1.0, 2.0, 4.0, 8.0, 16.0, 32.0, and 64 ppm of TNT. For this purpose, due to the low solubility of 2,4,6-trinitrotoluene in water, standard stock solutions of TNT were prepared in acetonitrile with a concentration of 10,000, 1000, and 100 ppm. The 0.1 M PBS was prepared by adding 51 mL of 0.2 M KH_2_PO_4_, 49 mL of 0.2 Na_2_HPO_4_, and 100 mL of demineralized water. The pH of solution was chosen based on our previous research [28]. For electrochemical measurements, the aliquot volume of standard stock solutions was added to 10 mL of PBS (see Table S1). The DPV parameters were firstly optimized. The obtained parameters for the DPV measurements were pulse height of 100 mV, pulse width of 70 ms, step height of − 10 mV, and step time of 1000 ms. The volume of the measured sample was equal to 2 mL. The investigated aqueous sample was not subjected to any purification process (it was directly placed in the electrochemical cell). The calibration curves were obtained by reading the maximum height of the current density peak for the selected reduction peak observed at − 0.4 V vs. Ag|AgCl|3 M KCl (using OriginLab software).

### Chemicals

2,4,6-Trinitrotoluene (99%) was obtained from Nitro-Chem JSC (Poland). HPLC-grade extra pure acetonitrile was used to prepare the 2,4,6-trinitrotoluene stock solution. All reagents were analytical grade and used without further purification.

## Result and discussion

### Morphology and electrochemistry of the composite electrodes

The top-view SEM image of the G-PLA-NDF-bottom electrode is displayed in Fig. 1c. The section of NDF was mechanically removed from electrode to expose the morphology of thermally tuned interface. The PLA exhibits clearly texture of top surface of NDF mirroring its morphology. Observed effect proves direct electric contact of both materials. Moreover, the developed polycrystalline morphology of NDF’s top surface increases additionally specific surface area of adhered G-PLA revealing high and continuous contact area. Additionally, the concentration of boron at the NDF top surface was found to be higher than that recorded for the bottom NDF surface [23, 27] providing efficient carrier transfer. Therefore, designed multi-layer electrode delivers enhanced electrochemical and electrical performance versus bare G-PLA.

The top exposed in SEM image NDF-bottom surface (i.e., G-PLA-NDF-bottom) reveals a smooth morphology with smaller grains thanks to use of Ta substrate during CVD synthesis [22, 27]. Magnified image of G-PLA-NDF cross section is shown in inset of Fig. 1c revealing tight adhesion and lack of interfacial gaps. Next, the minor, local variation of SEM brightness could be attributed to the varied surface potentials recorded at NDF and G-PLA or distinguished at high roughness regions.

The focused ion beam microfabricated cross section of the G-PLA-NDF electrode is presented in Fig. 1d. The NDF is fully integrated with G-PLA exhibiting uniform and continuous contact of both layers (see inset in Fig. 1d). The NDF fully encapsulates G-PLA resulting in effective electrode design, where G-PLA serves synergistically as highly conductive collector and flexible holding platform for brittle NDFs. Moreover, applied approach defines and stabilizes porous G-PLA structure manufactured by additive technique. The thermal treatment utilized for junction of NDF with G-PLA induced also PLA crystallinity transformation to the α one and improved homogeneity as previously reported by Liao et al. [29]. Furthermore, an extended molecular and morphologic analysis of bare boron-doped diamond nanosheet surfaces has been reported elsewhere [22, 23, 27].

**Fig. 2:**
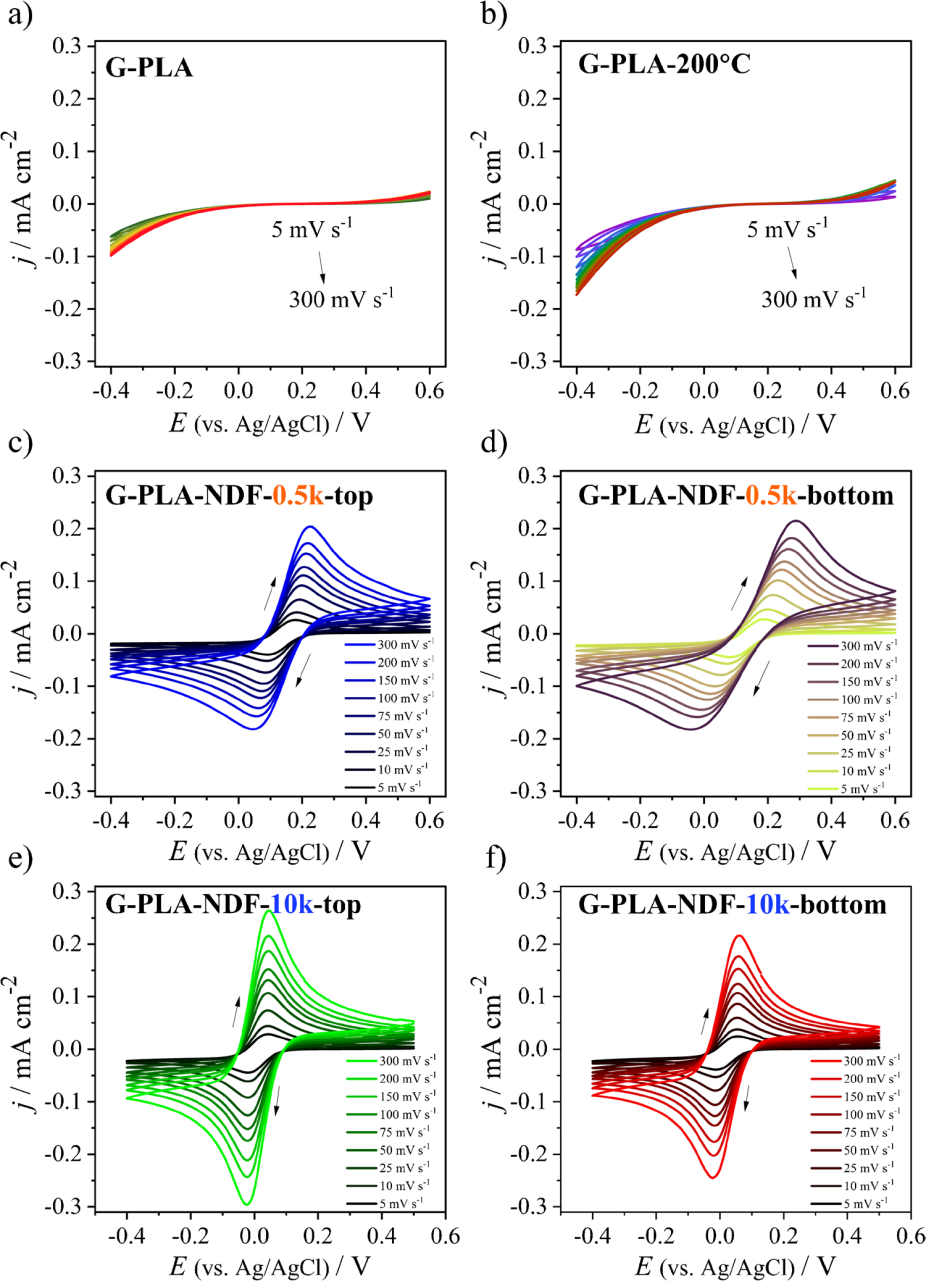
Cyclic voltammetry curves of (**a**) G-PLA, (**b**) G-PLA-200 °C, (**c**) G-PLA-NDF-0.5 k-top, (**d**) G-PLA-NDF-0.5 k-bottom, (**e**) G-PLA-NDF-10 k-top, and (**f**) G-PLA-NDF-10 k-top electrodes immersed in 1 mM K_3_[Fe(CN)_6_]/1 mM K_4_[Fe(CN)_6_] + 0.5 M Na_2_SO_4_ as a function of scan rates

The electrochemical behavior of printed graphene/poly(lactic acid) electrode was firstly studied using cyclic voltammetry in the presence of hexacyanoferrate (II/III) redox mediator (Fig. 2a). Polylactide is a polymer that shows poor conductivity at room temperature; thus, it inhibits the electron transfer at the electrode–electrolyte interface. As a result, the voltammogram of the G-PLA does not show the oxidation and reduction peaks of the [Fe(CN)_6_]^3−/4−^ redox system. Blocking the redox reaction is also visible after heating the electrode on a hot plate to 200 °C (G-PLA-200 °C) (see Fig. 2b).

The next step was electrochemical testing of the surface-modified G-PLA by nanodiamond foil (with different boron doping) graphene-PLA-NDF composites: G-PLA-NDF-0.5 k-bottom, G-PLA-NDF-0.5 k-top, G-PLA-NDF-10 k-bottom, and G-PLA-NDF-10 k-top (Fig. 2c–f). For composite electrodes, the appearance of the oxidation and reduction peaks of the [Fe(CN)_6_]^3−/4−^ redox mediator can be noticed. The CV curves vary depending on the type of foil used (0.5 k or 10 k) and on the side that is in contact with the electrolyte (top or bottom). An important electrochemical parameter proving the kinetics of the oxidation and reduction of the redox pair, and thus the possibility of electron transfer through the electrode, is the distance between the oxidation and reduction peak called the peak-to-peak separation (Δ*E*). There is a significant difference between the composite electrodes obtained using low-doped boron 0.5-k foil and the electrodes containing high-doped 10-k foil. With increasing the sweep rate, the Δ*E* significantly increases for the G-PLA-NDF-0.5 k electrodes from 105 to 310 mV for G-PLA-NDF-0.5 k-bottom, and from 88 to 172 mV for G-PLA-NDF-0.5 k-top (the detailed values of each peak-to-peak separation depending on the scan rate are collected in Table S2). The significant width of the peak-to-peak separation may be the first sign that the Faradaic reaction is electrochemically irreversible.

Therefore, the next step was to estimate the value of the parameter introduced by Matsuda and Ayabe Λ and heterogeneous electron transfer (HET) rate constant, which can help in classifying the type of electrochemical reaction (calculation method is described in Supplemental Information) [30, 31]. The results calculated for a sweep rate of 100 mV s^−1^ are gathered in Table 1.

**Table 1:** Comparison of electrochemical parameters of G-PLA-NDF composite electrodes (*ν* = 100 mV s^−^.^1^)

	G-PLA-NDF-0.5 k-bottom	G-PLA-NDF-0.5 k-top	G-PLA-NDF-10 k-bottom	G-PLA-NDF-10 k-top
*E*_*p,ox*_ (mV)	250	205	153	141
*j*_*p,ox*_ (μA cm^−2^)	154	148	150	181
*E*_*p,red*_ (mV)	19	72	83	78
*j*_*p,red*_ (μA cm^−2^)	− 133	− 134	− 154	− 186
Δ*E*_*p*_ (mV)	231	133	68	63
Λ	9.6 × 10^−2^	0.5	5.1	11.2
Ψ	-	0.3	2.9	6.3
*kº* (cm s^−1^)	5.20 × 10^−4^	2.80 × 10^−3^	2.8 × 10^−2^	6.1 × 10^−2^

According to the literature [32], the values of parameter Λ ranging from 0.096 to 11.2 indicate quasi-reversible Faradaic reactions. Thus, all G-PLA-NDF electrodes are characterized by a quasi-reversible nature of the [Fe(CN)_6_]^3−/4−^ redox system reaction. It should be noted, however, that the G-PLA-NDF-0.5 k-bottom composite is already on the borderline between the quasi-reversible and the irreversible reaction, as indicated by the Δ*E* value of over 200 mV, often considered the upper limit of the quasi-reversible charge transfer reaction.

Composite electrodes made using highly doped foil are characterized by significantly higher values of the Λ parameter. This is especially visible for the G-PLA-NDF-10 k-top electrode which is very close to the value Λ = 15, the limit of the reversible reaction. The G-PLA-NDF-10 k-top electrode is also characterized by the fastest HET rate constant of 6.1 × 10^−2^ cm s^−1^. Moreover, the G-PLA-NDF-10 k-top electrode shows a slight peak shift, only 8 mV, with an increasing scan rate from 61 mV (*ν* = 5 mV s^−1^) to 68 mV (*ν* = 300 mV s^−1^).

The *kº* value for G-PLA-NDF-0.5 k-bottom was calculated based on the formula for one-electron irreversible reactions (detail in Supplemental Information). The standard rate constant for the G-PLA-NDF-0.5 k-bottom electrode was 5.20 × 10^−4^ cm s^−1^. Furthermore, current densities recorded for composites obtained with the use of 0.5-k doped foil (G-PLA-NDF-0.5 k), regardless of which side of the diamond foil is in contact with the electrolyte, are definitely lower than in composites made with the use of 10-k highly doped diamond foil. For the sweep rate equal to 300 mV s^−1^, the current density recorded for the G-PLA-NDF-10 k-top electrode was 0.27 mA cm^−2^, whereas for G-PLA-NDF-0.5 k-bottom it was 0.22 mA cm^−2^.

In Figure S1, the dependence of the anodic and cathodic peak current on the square root of the sweep rate is shown (prepared based on diagrams shown in Fig. 2). For each G-PLA-NDF electrode, a linear relationship between *i*_*p*_ and *ν*^1/2^ can be seen (obtained for both oxidation and reduction processes). For the G-PLA-NDF-10 k electrodes, the slope of the regression line is higher compared to the G-PLA-NDF-0.5 k electrodes (see Table S3). Moreover, the coefficient of determination (*R*^2^) achieves values in the range of 0.9996 to 0.9999 for electrodes with highly doped foils.

As a result of the CV measurements, it can be concluded that the electrodes in which the bottom side of the nanodiamond foil is in contact with the electrolyte exhibit generally deteriorated electrochemical behavior. This may be related to the lower boron concentration and higher conductivity of the bottom side of nanodiamond foils, as shown in our previous research [22]. Moreover, appropriately high doping of the diamond with boron is a key parameter that directly influences the electrochemical response of the material. The results are confirmed in other literature reports [33]. Therefore, for further research, only composites containing highly doped diamond foils were applied.

The electrochemical impedance study was carried out to further investigate the electrochemical kinetic parameters. Figure S2 shows the Nyquist plot of the impedance spectra. For better interpretations of the EIS results, the spectra were fitted using an Electrical Equivalent Circuit (EEC) shown in Fig. S3 using a ZSimpWin analyzer. All impedance results were matched with one EEC, which was selected according to the fitting of the composite electrode spectra. The EEC consists of four elements: electrolyte resistance *R*_*e*_, charge-transfer resistance *R*_ct_, constant phase element *CPE*, and Warburg impedance *W.* The CPE element is defined as *Z*_CPE_ = 1/*Q*(*jω*)^*n*^, where *ω* (rad s^−1^) reflects the angular frequency, *j* corresponds to the imaginary number, and *n* and *Q* are the parameters of the CPE element. The constant phase element is often used for fitting the impedance spectra of diamond electrodes due to its frequency-dependent capacitance [34]. The fitted parameters are gathered in Table 2.

**Table 2:** List of values of elements calculated from the EEC for graphene-PLA electrodes and the composite electrode with highly doped nanodiamond foil

	G-PLA	G-PLA-200 °C	G-PLA-NDF-10 k-top	G-PLA-NDF-10 k-bottom
*R*_*e*_ (Ω cm^2^)	23.2	7.0	8.1	8.3
*Q* (Ω^−1^ cm^−2^ s^*n*^)	9.6 × 10^−8^	3.8 × 10^−7^	1.7 × 10^−5^	1.1 × 10^−5^
*n*	0.97	0.95	0.87	0.86
*R*_ct_ (Ω cm^2^)	8.50 × 10^5^	2.52 × 10^5^	17	15
*W* (Ω^−1^ s^0.5^ cm^−2^)	-	-	1.9 × 10^−3^	1.7 × 10^−3^
*C*_eff_ (µF cm^−2^)	0.1	0.2	4	2.6
*kº* (cm s^−1^)	3.1 × 10^−7^	1.1 × 10^−6^	1.6 × 10^−2^	1.8 × 10^−2^

The impedance spectra recorded on the G-PLA-NDF-10 k electrodes have a different shape than the electrodes without the nanodiamond foils (G-PLA and G-PLA-200 °C). In the high-frequency region, the impedance spectra recorded on the G-PLA-NDF-10 k samples form a small semi-circle, whereas at the low-frequency region, there is a straight line tilted approximately 45° to the *X*-axis. Such a shape suggests a diffusion-limited process for electron transfer reaction. The observations are different for the G-PLA and G-PLA-200 °C electrodes, for which the entire spectrum is semi-circle-shaped. The diameter that this semi-circle reaches is the value of the charge-transfer resistance. The *R*_ct_ value is 15 Ω cm^2^, 17 Ω cm^2^, 2.52 × 10^5^ Ω cm^2^, and 8.50 × 10^5^ Ω cm^2^ for G-PLA-NDF-10 k-bottom, G-PLA-NDF-10 k-top, G-PLA-200 °C, and G-PLA, respectively. Hence, it can be concluded that the charge transfer reaction controls the electrochemical process taking place on the G-PLA and G-PLA-200 °C electrodes. The decrease of the *R*_ct_ value after heating the G-PLA electrode, and thus a slight increase in conductivity, may result from the displacement of conductive graphene particles towards the electrode surface.

The results of *k*^*o*^ obtained for the G-PLA-NDF-10 k foils are of the same order of magnitude as the constants previously calculated by Nicholson’s method and are 1.6 × 10^−2^ cm s^−1^ for G-PLA-NDF-10 k-top, and 1.8 × 10^−2^ cm s^−1^ for G-PLA-NDF-10 k-bottom. The values of effective electric double-layer capacitance calculated for G-PLA-NDF-10 k are in the range of 2.6 to 4 µF cm^−2^, which are typical for the diamond phase [35–37].

Furthermore, we compared the electrochemical behavior of the composite electrodes in which widely available conductive copper foil was used instead of G-PLA. For this purpose similar CV and EIS measurements were conducted using Cu-NDF-10 k-top and Cu-NDF-10 k-bottom. The values of peak-to-peak separation are collected in Table S2, while the impedance spectra are shown in Fig. S4. The electric parameters calculated for EEC are listed in Table S4. It was found that the electrodes with copper foil exhibit wider Δ*E* values and a higher shift of [Fe(CN)_6_]^3−/4−^ oxidation and reduction peak maxima with the growing scan rate. Moreover, the charge transfer resistance values recorded on Cu-NDF-10 k foils are almost twice as large and reach *R*_ct_ of 26 Ω cm^2^ and 32 Ω cm^2^ for Cu-NDF-10 k-bottom and Cu-NDF-10 k-top, respectively. The potential barrier observed at the copper could be much larger because of the acrylic conductive adhesive at the utilized copper tape, which could additionally increase the work function of this collector. Moreover, the application of the graphene-PLA composite creates an enhanced current collector in comparison to solid copper, which suffers from the continuity of its mechanical interface with NDF. Solid copper forms rather multi-point contact with hot spots than the continuous interface. It is also known that BDD shows larger cross-sectional conductivity than lateral one due to heterogenous columnar structure, where the continuous and high area contact plays an even more important role. Furthermore, graphene shows a work function of ∼4.6 eV but provides a low contact resistance [38], while bare copper exhibits a slightly larger work function of 4.7 eV.

The main advance of graphene-PLA composite approach is that melted G-PLA reforms its interface to the pattern of diamond flake morphology, which is polycrystalline with heterogeneous conductivity behavior. It allows for homogenous current collection integrating effectively signals from the NDF electrode surface, which is also confirmed by the electrochemical properties. Therefore, it can be concluded that the novel G-PLA-NDF-10 k composite electrodes could useful for various detection approaches.

### TNT detection

**Fig. 3:**
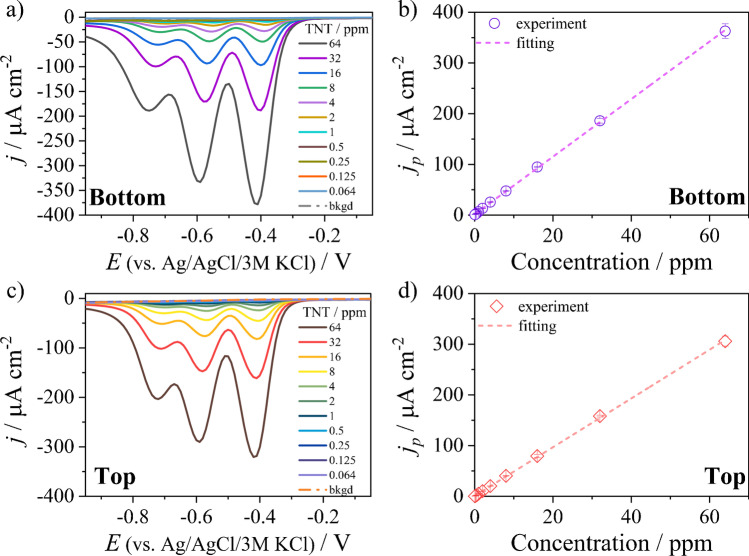
Detection results of 2,4,6-trinitrotoluene obtained by the DPV technique recorded on (**a**) G-PLA-NDF-10 k-bottom and (**c**) G-PLA-NDF-10 k-top; calibration curve performed based on the peak occurring at a potential of about − 0.4 V vs. Ag|AgCl|3 M KCl of (**b**) G-PLA-NDF-10 k-bottom and (**d**) G-PLA-NDF-10 k-top (the error bar indicates the standard deviation of the results for the measured samples)

The electrodes exhibiting enhanced electrochemical behavior were used to detect the widely used explosive compound, 2,4,6-trinitrotoluene (TNT) [39–41], utilizing the electrochemical technique of differential pulse voltammetry (DPV). The DPV investigations were carried out in 0.1 M PBS (pH = 6.8). The voltammogram shown in Fig. 3a and c shows the presence of three well-separated cathode peaks at − 0.41 V, − 0.59 V, and − 0.76 V vs. Ag|AgCl|3 M KCl related to the reduction of the three nitro groups of the TNT compound.

The calibration curves (*y* = *ax* + *b*) are presented in Fig. 3b and d. There is a wide linear relationship between the peak current density and the analyte concentration in the wide concentration range of 0.064 ppm and 64 ppm for both the G-PLA-NDF-10 k-bottom and G-PLA-NDF-10 k-top electrodes (*R*^2^ = 0.9999, *N* = 3). The regression lines are expressed by the formulae *j*_*p*_ [μA cm^−2^] = (4.807 ± 0.027) *c*_TNT_ [ppm] + (0.676 ± 0.606) and *j*_*p*_ [μA cm^−2^] = (5.678 ± 0.026) *c*_TNT_ [ppm] + (1.591 ± 0.590) for G-PLA-NDF-10 k-top and G-PLA-NDF-10 k-bottom, respectively. The sensitivity of TNT detection can then be determined on the basis of the slope of the regression line, which in the case of the G-PLA-NDF-10 k-bottom electrode was equal to 1.290 ± 0.006 μA μM^−1^ cm^−2^ (5.678 ± 0.026 μA ppm^−1^ cm^−2^) and 1.0925 ± 0.006 μA μM^−1^ cm^−2^ (4.807 ± 0.027 μA ppm^−1^ cm^−2^) for the G-PLA-NDF-10 k-top electrode.

The G-PLA-NDF composite electrodes showed good repeatability. The RSD value estimated on currents density obtained by detecting 8 ppm TNT from the solution using five electrodes was 6.4%. Spiked samples at different concentrations levels (within the calibration curve range) gave recoveries in the range from 93.8 to 106.4%. The limit of detection (LOD) was evaluated from LOD = 3*σ*_bl_/*a* [42–44], where *σ*_bl_ reflected the standard deviation of a blank and “*a*” corresponds to the slope of the calibration curve. The calculated LOD values are equal to 87 ppb (0.383 μM) and 315 ppb (1.39 μM) for G-PLA-NDF-10 k-bottom and G-PLA-NDF-10 k-top, respectively. According to the BTAG Freshwater Screening Benchmarks published by United States Environmental Protection Agency [45], the ecological screening value of TNT is 0.44 μM. Thus, the sensor G-PLA-NDF-10 k-bottom could be used for screening environmental tests.

Table 3 presents a comparison of 2,4,6-trinitrotoluene detection performances from the present and previously reported electrochemical sensors depending on the electrode used.

**Table 3:** An overview on recently reported nanomaterial-based electrochemical methods for the determination of 2,4,6,-trinitrotoluene

Electrode material	Linear range (µM)	Limit of detection (µM)	Ref
MWCNT-GC	0.44–4.4	0.00264	[46]
B-NCD	0.0088–1.76	0.044	[47]
GC/P(Cz-*co*-ANI)-Au_nano_	0.44–4.4	0.11	[42]
B:DGNW	0.22–8.81	0.321	[28]
G-PLA-NDF-10 k-bottom	0.282–282	0.383	This work
Ag/CS-G/GCE	4.4–484	2.64	[48]
G-FL	4.4–66	4.4	[49]
G-LiClO_4_	17.6–88.1	29.7	[50]

*MWCNT-GC* multi-wall carbon nanotube-modified glassy carbon**,** *B-NCD* boron**-**doped nanocrystalline diamond**,** *GC/P(Cz-co-ANI)-Au*_*nano*_ gold nanoparticle/poly(carbazole-aniline) film**-**modified glassy carbon**,** *B:DGNW* boron-doped diamond/graphene nanowall electrodes**,** *Ag/CS-G/GCE* glassy carbon electrode modified with graphene with carboxylic sodium groups and functionalized with Ag**,** *G-FL* few-layer graphene nanoribbons**,** *G-LiClO*_*4*_ electrochemically exfoliated graphene in LiClO_4_

The G-PLA-NDF-10 k-bottom electrode shows slightly greater electrocatalytic activity towards TNT determination than G-PLA-NDF-10 k-top, which is evidenced by both the detection limit and the sensitivity of the measurement. This may be due to the difference in the amount of the non-diamond carbon (NDC) phase. As showed previously [22], the bottom side of nanodiamond foil is characterized by a higher amount of NDC. According to the literature, the 2,4,6-trinitrotoluene electro-reduction delocalized electron in *sp*^2^ carbon promotes the adsorption process of the TNT compound with an electron-deficient aromatic ring via π-π interactions [28, 50]. The *sp*^2^ carbon-dependent mechanism was also confirmed by cyclic voltammetry of standard boron-doped diamond electrodes (see Figure S5). For this purpose, we deposited, using the microwave plasma-assisted chemical vapor deposition process, a standard boron-doped diamond electrode (BDD) on a *p*-type silicon substrate (the detailed procedure can be found here [51]) being superficially analogous to NDF. Generally, the chemical vapor process induces the formation of non-diamond carbon phases simultaneously with the *sp*^3^-rich BDD phase growth. Those degenerated non-diamond *sp*^2^ phases are often an undesirable outcome [33]. Thus, we conducted extended treatment of the BDD surface with hot aqua regia (HNO_3_:HCl/1:3, v-v), then with a hot “piranha” solution (H_2_O_2_:H_2_SO_4_/1:3, v-v) at 90 °C followed by hydrogen plasma exposure (1000-W microwave power and 300 sccm of H_2_ gas flow for 10 min). As a result, the BDD surface was made predominantly hydrogen-terminated (BDD-H). The as-grown BDD electrodes revealed three well-separated peaks of TNT reduction in the CV curves, attributed to the *sp*^2^-tuned process. The electrocatalytic process of the treated BDD-H was less efficient, which resulted in the widening of peaks observed at lower potentials.

Thus, the *sp*^2^ phases at the NDF surface could efficiently mediate the charge transport at both the G-PLA/NDF and electrode/electrolyte interfaces. Next, *sp*^2^ phases exhibit a similar electronic band structure to G-PLA, improving the contact and electron transport creating the synergistic effect of high electrochemical performance and low resistivity delivered jointly with the *sp*^3^ phase in highly boron doped diamond foils. We recognized the G-PLA-NDF electrode as an enhancement of the standard 3D-printed graphene-rich surface revealing electrochemical activation observed in both CV and EIS plots.

The mechanism of interfacial charge transport is complex and its understanding needs deeper insights allowing for further improvement in terms of sensitivity and LOD by tuning, i.e., the concentration or shape of the NDF flakes at the G-PLA surfaces. Nevertheless, the proposed approach is an environmentally friendly and relatively simple method providing a practical strategy for improving the electroanalytical response of G-PLA electrodes for a variety of applications, where light weight and flexible electrode of a specific shape is required. However, it should be emphasized that inside the complex environmental samples containing other electroactive species that are reducing in the range of − 0.3 to − 0.9 V, the proposed electrodes will show high sensitivity but lower selectivity towards TNT. Unmodified carbon-based electrode material will detect a wide range of explosive compounds such as, for example, TNT, 2,4-dinitrotoluene (2,4-DNT), 2,6-dinitrotoluene (2,6-DNT), 4,4′,5,5′-tetranitro-1H,1′H-2,2′-biimidazole, 2,4,6-trinitroanisole, and 1,3,5,7-tetranitro-1,3,5,7-tetrazocane (HMX) [40, 42, 47, 52]. It should be noted that TNT rarely occurs as a single explosive in the aquatic environment, for example, during the military conflicts that took place in the twentieth century often used blasting mixtures consisting of TNT and RDX. Moreover, it is possible that in an aqueous medium TNT will be reduced to a form containing a smaller amount of nitro groups, such as DNT. Thus, the wide range of responses to explosive compounds of diamond electrodes could be also an enormous advantage when used to monitor the environment for the presence of nitroexplosive compounds. The difficulty will be in distinguishing these compounds from one another. In the case of some explosive compounds with a similar chemical structure but with a different amount of nitro groups (e.g., 2,4-DNT, 2,6-DNT compared to TNT) it is possible to distinguish between compounds (electrochemical reduction of DNT containing two nitromoieties will generate two reduction peaks, while in the case of TNT it will be three reduction peaks) [47]. Nevertheless, with the help of electrochemical sensors, it is possible to measure a large number of real samples in a short time and at a relatively low cost. Hence, by using G-PLA-NDF electrodes, it is possible to carry out field screening tests and select samples that would be sent for further diagnostics (e.g., for chromatographic measurements).

## Conclusions

We proposed a novel approach of freshly printed graphene-PLA plates as a conductive adhesive for diamond flakes for electrochemical sensor. The direct contact of the NDF and G-PLA created a rigid and mechanically stable electrode interface with enhanced local tunneling in the diamond/graphene junction configuration. It should be emphasized that the melted G-PLA reforms its interface to the pattern of diamond flake morphology, which allows for homogenous current collection integrating sensing signals from the NDF electrode surface. Furthermore, since diamond flakes are flexible and diamond thin nanostructures could be bent up to 10%, the proposed approach could be used also for sensors working in flexible modes (i.e., [27]).

A variety of standard contacts could be applied for electrochemical sensors instead of G-PLA, i.e., conductive carbon or copper adhesives, conductive carbon, or silver pastes. Nevertheless, compared to the printed G-PLA they are relatively costly and not convenient for scaling sensor fabrication process. Moreover, the presented method can be applied also using other modifiers (instead of NDF), e.g., diamond powders and carbon nanotubes, which allows the adaptation of the electrode material to the specific application.

The highly doped G-PLA-NDF-10 k composite electrode could be successfully used as an electrochemical sensor. The bottom side of G-PLA-NDF-10 k electrode resulted in LOD values equal to 87 ppb of TNT and wide linearity range (0.064–64 ppm), manifesting high performance for, i.e., portable devices, flexible forensic, or wearable sensors. Moreover, the wide range of responses to explosive compounds of diamond electrodes enables the use of these electrodes to monitor the environment against a family of explosive compounds in a short time and a relatively low cost.

The next step could be miniaturization of G-PLA-NDF electrodes, where bottom-up-shaped diamond flakes (by selective seeding) could be positioned by a robot at the 3D printer placing them on a single graphene-PLA plate or the multi-sensor plate formed from two different filaments (conductive and isolating) in the various shapes and sizes.

## Supplementary Information

Below is the link to the electronic supplementary material.

Supplementary file1 (PDF 816 KB)
