# Supplementary material for: Conductive printable electrodes tuned by boron-doped nanodiamond foil additives for nitroexplosive detection
Source: Mikrochim Acta. 2022 Jul 5;189(8):270. doi: 10.1007/s00604-022-05371-w (PMC9255478; doi:10.1007/s00604-022-05371-w)
Supplement: Supplementary file 1 — Supplementary file1 (PDF 816 KB) [file 604_2022_5371_MOESM1_ESM.pdf]

Supplemental Information

Conductive Printable Electrodes Tuned by Boron-Doped Nanodiamond Foils Additives for Nitroexplosive Detection

Anna Dettlaff[a b,], Michał Rycewicz[b], Mateusz Ficek[b], Aleksandra Wieloszyńska[b], M. Szala[c], Jacek Ryl[d], Robert Bogdanowicz[b]*

[a]Gdańsk University of Technology, Faculty of Chemistry, Department of Energy Conversion and Storage, 11/12 Narutowicza St., 80-233 Gdańsk, Poland

[b]Gdańsk University of Technology, Faculty of Electronics, Telecommunications and Informatics, Department of Metrology and Optoelectronics, 11/12 Narutowicza St., 80-233 Gdańsk, Poland

[c]Military University of Technology, S. Kaliskiego 2, 00-908 Warsaw, Poland

[d]Gdańsk University of Technology, Institute of Nanotechnology and Materials Engineering and Advanced Materials Center, 11/12 Narutowicza St., 80-233 Gdańsk, Poland

[*]Corresponding Author.
E-mail: anna.dettlaff@pg.edu.pl

Preparation of diamond foil

Nanodiamond foils were grown in a Microwave Plasma Enhanced Chemical Vapor Deposition reactor with a frequency of 2.45 GHz (SEKI Technotron AX5400S, Japan) on mirror-polished tantalum foils (Sigma-Aldrich Chemie, 0.4 mm thick). All substrates were cleaned in acetone and 2-propanol for 10 minutes. After cleaning, the substrates were ultrasonically seeded for 30 minutes in a water suspension consisting of nanodiamond particles of 4–7 nm in size. The substrate temperature, plasma microwave power, and pressure inside the chamber were 500°C, 1100 W, and 50 Torr, respectively. The CH_4/H_2 molar ratio of the mixture was 1%.

Preparation of the investigated TNT solutions

Table S1. Preparation of the investigated TNT solutions.

TNT stock solution concentration / ppm	Volume of stock solution added to the 10 ml PBS / µL	TNT concentration in PBS solution / ppm
**10 000**	81.4	64
	40.7	32
	20.4	16
	10.2	8
**1 000**	50.9	4
	25.4	2
	12.7	1
	6.4	0.5
**100**	31.8	0.250
	15.9	0.125
	8.0	0.064

Table S2. Peak-to-peak separation recorded for G-PLA-NDF electrode in the electrochemical mediator 1 mM $[Fe(CN)_6]^{3-}$ / 1 mM $[Fe(CN)_6]^{4-}$.

	ΔE_p / mV					
**ν / mV s^{-1}**	**G-PLA-NDF-0.5k-bottom**	**G-PLA-NDF-0.5k-top**	**Cu-NDF-10k-bottom**	**Cu-NDF-10k-top**	**G-PLA-NDF-10k-bottom**	**G-PLA-NDF-10k-top**
**5**	105	88	68	61	68	61
**10**	129	92	74	65	65	61
**25**	160	106	86	73	66	61
**50**	193	116	98	80	69	62
**75**	213	125	102	85	66	62

**100**	231	133	110	90	68	64
**150**	259	145	117	98	73	67
**200**	279	158	125	102	80	65
**300**	310	172	143	110	80	68

Calculation of electrochemical parameters

The value of parameter Λ was calculated using the Eq. (1) [1–3]:

$$\Lambda = k° \cdot \sqrt{\frac{R \cdot T}{F \cdot D \cdot \nu \cdot n}}, \tag{1}$$

where k^o [cm s^{-1}] corresponds to heterogeneous electron transfer (HET) rate constant, R is the molar gas constant (8.314 J mol^{-1} K^{-1}), T reflects the temperature (298 K), F is the Faraday constant (96,485 C), D is the diffusion coefficient ($7.6 \cdot 10^{-6}$ cm^2 s^{-1}) [3], ν [V s^{-1}] is the scan rate, and n is the number of electrons (1).

The HET rate constant necessary to calculate the previous Λ parameter was estimated using the Eq. (2) [1].

$$k° = \psi \cdot \sqrt{\frac{\pi \cdot D \cdot \nu \cdot F \cdot n}{R \cdot T}}, \tag{2}$$

where ψ is a kinetic parameter estimated based on the ΔE value by the Nicholson method (Table below) [4].

Kinetic parameter depending on the peak-to-peak separation value for 1-electron redox reaction (assumption α = 0.5) [4].

ψ	ΔE_p / mV
**20**	61
**7**	63
**6**	64
**5**	65
**4**	66
**3**	68
**2**	72
**1**	84
**0.75**	92
**0.5**	105
**0.35**	121
**0.25**	141
**0.1**	212

The k^o value for G-PLA-NDF-0.5k-bottom was calculated based on the formula for one-electron irreversible reactions (Eq. (3)) [1]:

$$E_p = E^{o\prime} - \frac{R\cdot T}{\alpha\cdot F}\left[0.78 - ln\frac{D_o^{1/2}}{k^{\circ}} + 0.5ln\frac{\alpha\cdot F\cdot \nu}{R\cdot T}\right] \tag{3}$$

where $E^{o\prime}$ [V] corresponds to the formal potential, D_o reflects the diffusion coefficient of oxidized species, and α is the transfer coefficient, equal to 0.21, previously calculated from Eq. (4):

$$i_p = 2.99\cdot 10^5\cdot\sqrt{\alpha}\cdot A\cdot c\cdot D^{\frac{1}{2}}\cdot \nu^{\frac{1}{2}} \tag{4}$$

where A [cm^2] is the electroactive surface area of the electrode (in this case we have assumed that the geometric area of the electrode is equal to the electroactive area), and c [mol cm^{-3}] is the concentration.

The HET rate constant was also calculated from the impedance spectra according to Eq. (5) [5, 6].

$$k^{\circ} = \frac{R\cdot T}{n^2\cdot F^2\cdot A\cdot C\cdot R_{ct}} \tag{5}$$

From the EEC fitting, assuming the surface distribution of the frequency capacitance dispersion, it is also possible to estimate the effective capacitance of the layer according to Eq. (6) [7].

$$C_{eff} = Q^{1/n}\cdot R_e^{1-n/n} \tag{6}$$

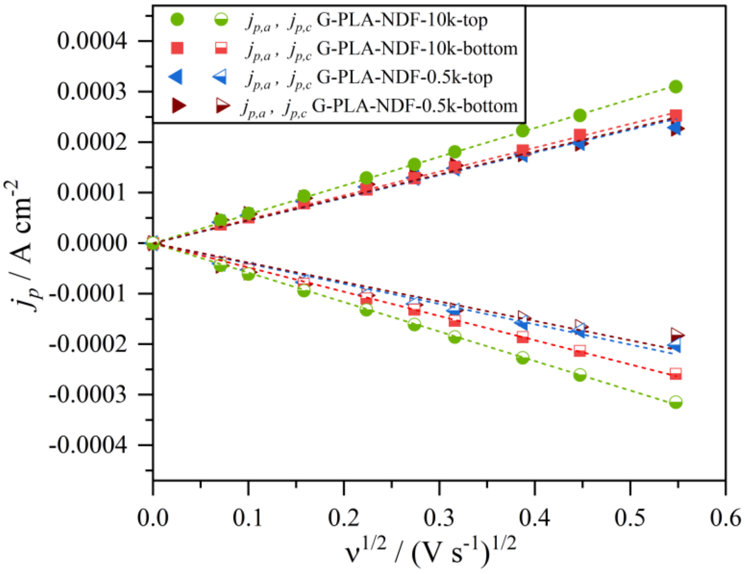

Figure S1. Dependence of the peak current on the scan rate obtained for G-PLA-NDF electrodes.

Table S3. The slope of the regression lines and the coefficients of determination estimated for G-PLA-NDF electrodes calculated based on linear fitting lines shown in Fig. 3.

	Oxidation		Reduction	
	Slope / A cm^{-2} $V^{-1/2}$ $s^{1/2}$	R^2	Slope / A cm^{-2} $V^{-1/2}$ $s^{1/2}$	R^2
**G-PLA-NDF-10k-top**	$5.702\cdot10^{-4}$ ($\pm$ $0.026\cdot10^{-4}$)	0.9999	$-5.832\cdot10^{-4}$ ($\pm$ $0.024\cdot10^{-4}$)	0.9998
**G-PLA-NDF-10k-bottom**	$4.728\cdot10^{-4}$ ($\pm$ $0.034\cdot10^{-4}$)	0.9998	$-4.803\cdot10^{-4}$ ($\pm$ $0.033\cdot10^{-4}$)	0.9996
**G-PLA-NDF-0.5k-top**	$4.491\cdot10^{-4}$ ($\pm$ $0.101\cdot10^{-4}$)	0.9977	$-4.017\cdot10^{-4}$ ($\pm$ $0.121\cdot10^{-4}$)	0.9919
**G-PLA-NDF-0.5k-bottom**	$4.543\cdot10^{-4}$ ($\pm$ $0.139\cdot10^{-4}$)	0.9958	$-3.847\cdot10^{-4}$ ($\pm$ $0.178\cdot10^{-4}$)	0.9811

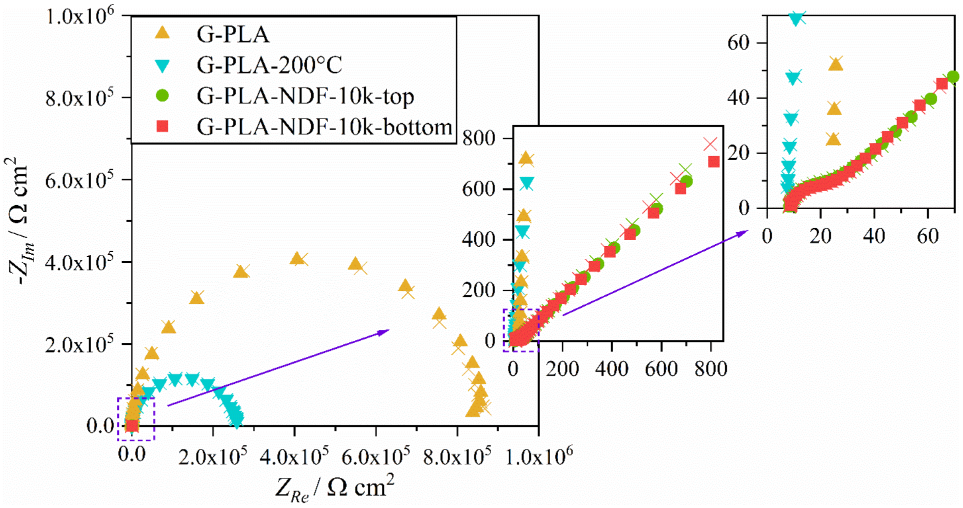

Figure S2. Nyquist plot of impedance spectra recorded for chosen electrodes at the formal potential.

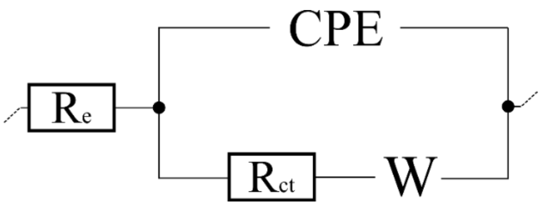

Figure S3. Electric equivalent circuit used for fitting.

Table S4. Selected electric parameters calculated for Cu-NDF-10k-top and Cu-NDF-10k-bottom electrodes on the basis of $R_e(CPER_{ct}W)$ equivalent circuit.

	**Cu-NDF-10k-top**	**Cu-NDF-10k-bottom**
R_e / Ω cm^2	34.3	8.1
CPE / Ω^{-1} cm^{-2} s^n	$1.6 \cdot 10^{-5}$	$5.9 \cdot 10^{-6}$
n	0.95	0.96
R_{ct} / Ω cm^2	32	26
W / Ω^{-1}s$^{0.5}$cm^{-2}	$3.9 \cdot 10^{-3}$	$2.6 \cdot 10^{-3}$
C_{eff} / μF cm^{-2}	11.0	3.8

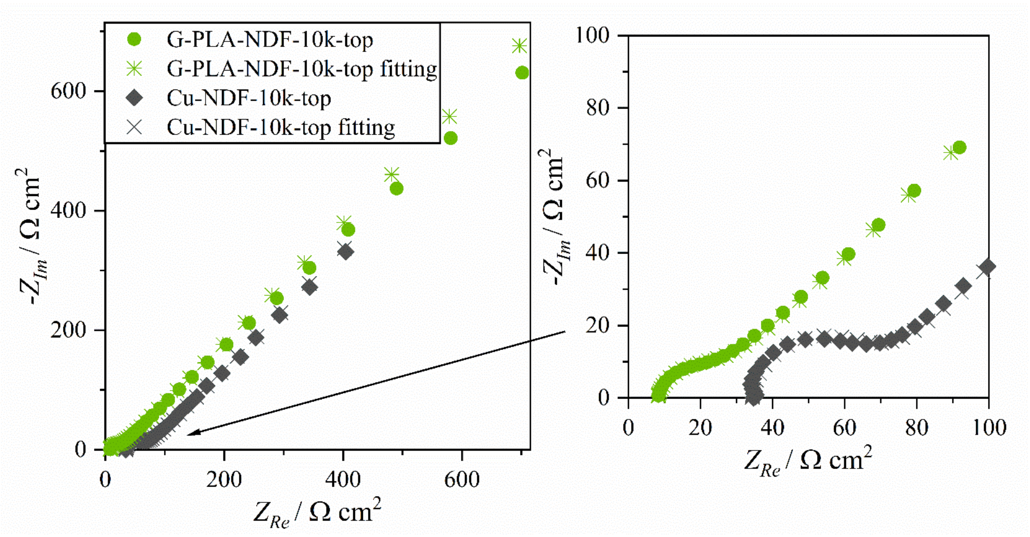

Figure S4. Nyquist plot of impedance spectra recorded on G-PLA-NDF-10k-top and Cu-NDF-10k-top in 1 mM $K_3[Fe(CN)_6]$/ 1 mM $K_4[Fe(CN)_6]$ + 0.5 M Na_2SO_4.

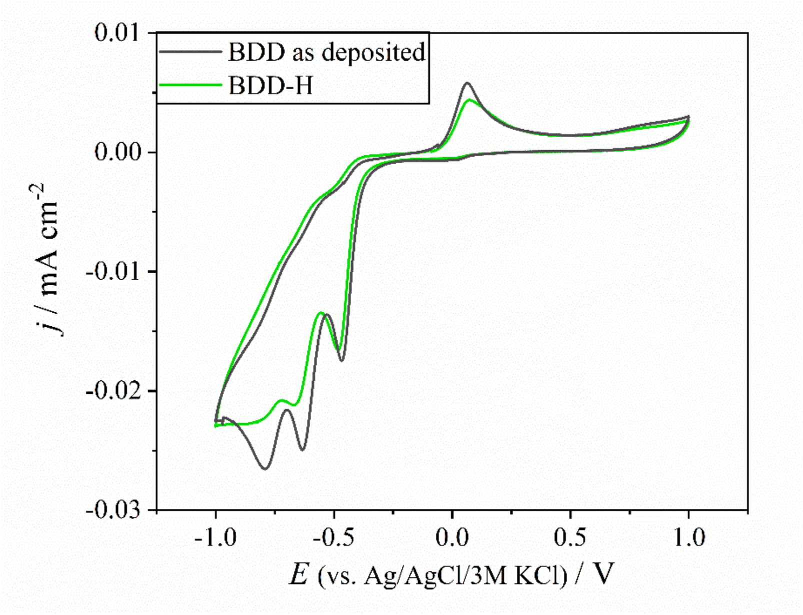

Figure S5. CV curves recorded on as deposited BDD and post-treated BDD terminated by hydrogen in 0.1 M PBS with 8 ppm of TNT.

1. Zoski CG (2007) Handbook of Electrochemistry. Elsevier
2. Compton RG, Banks CE (2018) Understanding Voltammetry. World Scientific
3. Bard AJ, Faulkner LR (2001) Electrochemical Methods. Fundamentals and Appliactions., Second. John Wiley @ Sons, INC.
4. Nicholson RS (1965) Theory and Application of Cyclic Voltammetry fm Measurement of Electrode Reaction Kinetics. Anal Chem 37:1351–1355
5. Dettlaff A, Sobaszek M, Klimczuk T, Bogdanowicz R (2021) Enhanced electrochemical kinetics of highly-oriented (111)-textured boron-doped diamond electrodes induced by deuterium plasma chemistry. Carbon N Y 174:594–604. https://doi.org/10.1016/j.carbon.2020.11.096
6. Krishnaveni P, Ganesh V (2021) Electron transfer studies of a conventional redox probe in human sweat and saliva bio - mimicking conditions. Sci Rep 1–13.

https://doi.org/10.1038/s41598-021-86866-z

7. Hirschorn B, Orazem ME, Tribollet B, et al (2010) Determination of effective capacitance and film thickness from constant-phase-element parameters. Electrochim Acta 55:6218–6227. https://doi.org/10.1016/j.electacta.2009.10.065
